# Serotype Distribution in Non-Bacteremic Pneumococcal Pneumonia: Association with Disease Severity and Implications for Pneumococcal Conjugate Vaccines

**DOI:** 10.1371/journal.pone.0072743

**Published:** 2013-08-23

**Authors:** Thomas Benfield, Marlene Skovgaard, Henrik Carl Schønheyder, Jenny Dahl Knudsen, Jette Bangsborg, Christian Østergaard, Hans-Christian Slotved, Helle Bossen Konradsen, Reimar Wernich Thomsen, Lotte Lambertsen

**Affiliations:** 1 Department of Infectious Diseases, Copenhagen University Hospital, Hvidovre, Denmark; 2 Clinical Research Centre, Copenhagen University Hospital, Hvidovre, Denmark; 3 Department of Clinical Medicine, Faculty of Health Sciences, University of Copenhagen, Copenhagen, Denmark; 4 Department of Clinical Epidemiology, Aarhus University Hospital, Aarhus, Denmark; 5 Department of Clinical Microbiology, Aalborg University Hospital, Aalborg, Denmark; 6 Department of Clinical Microbiology, Copenhagen University Hospital, Hvidovre, Denmark; 7 Department of Clinical Microbiology, Copenhagen University Hospital, Herlev, Denmark; 8 Statens Serum Institut, Copenhagen, Denmark; Centers for Disease Control & Prevention, United States of America

## Abstract

**Background:**

There is limited knowledge of serotypes that cause non-bacteremic pneumococcal pneumonia (NBP). Here we report serotypes, their associated disease potential and coverage of pneumococcal conjugate vaccines (PCV) in adults with NBP and compare these to bacteremic pneumonia (BP).

**Methods:**

Adults with pneumonia and *Streptococcus pneumoniae* isolated from the lower respiratory tract or blood were included 1 year in a population-based design in Denmark. Pneumonia was defined as a new infiltrate on chest radiograph in combination with clinical symptoms or elevated white blood count or plasma C-reactive protein. All isolates were serotyped using type-specific pneumococcal rabbit antisera. All values are medians with interquartile ranges.

**Results:**

There were 272 cases of NBP and 192 cases of BP. Ninety-nine percent were hospitalized. NBP and BP cases were of comparable age and sex but NBP cases had more respiratory symptoms and less severe disease compared to BP cases. In total, 46 different serotypes were identified. Among NBP cases, 5 serotypes accounted for nearly a third of isolates. PCV10 and -13 types covered 17% (95% confidence interval (CI): 11-23%) and 34% (95% CI: 25-43%) of NBP isolates, respectively. In contrast, the five most frequent serotypes accounted for two-thirds of BP isolates. PCV10 and -13 types covered 39% (95% CI: 30-48%) and 64% (95% CI: 48-79) of BP isolates, respectively. More severe NBP disease was associated with infection with invasive serotypes while there was an inverse relationship for BP.

**Conclusions:**

Only a third of cases of adult non-bacteremic pneumococcal pneumonia would potentially be preventable with the use of PCV13 and just one sixth of cases with the use of PCV10 indicating that PCVs with increased valency are needed to increase vaccine coverage for NBP in adults. PCV13 could potentially prevent two-thirds of adult bacteremic pneumococcal pneumonia.

## Introduction

Pneumonia is a leading cause of morbidity and mortality globally. Annual rates of hospitalization for community-acquired pneumonia (CAP) range from 2 to 4 per 1000 adult population in Europe [1,2]. More than half of pneumonia cases requiring hospitalization are believed to be caused by infection with *Streptococcus pneumoniae* [3]. Use of pneumococcal conjugate vaccines (PCV) for children has significantly reduced their rates of bacteremic and perhaps also non-bacteremic pneumococcal disease [4–6] but the efficacy of PCV for immunocompetent adults remains unknown. Data from a predominantly HIV-infected population, however, indicated that PCV7 was highly efficacious in preventing adults from reinfection with *S. pneumoniae* but the study did not distinguish between bacteremic and non-bacteremic pneumonia [7]. Protection conferred by the 23-valent polysaccharide vaccine (PPV23) is controversial for bacteremic pneumonia and doubtful for non-bacteremic pneumonia [8]. Further, the specificity of the vaccine is serotype dependent and therefore the protection it may confer is influenced by geographical and temporal changes in serotype distribution.

The pneumococcus is encapsulated by polysaccharide that protects it from host immune responses and is an important virulence factor. Variation of the capsule results in more than 90 different serotypes [9]. Poor outcome from pneumococcal disease is related to serotypes with high carriage rates, low invasiveness and heavy encapsulation [10–12]. While there is an abundance of data relating to invasive pneumococcal serotype distribution [13,14] there are very few studies that report serotype distribution of non-bacteremic pneumococcal pneumonia [15,16].

The current study was undertaken in order to establish the distribution of pneumococcal serotypes that cause non-bacteremic pneumonia (NBP), their association with disease severity and to compare them to cases of bacteremic pneumococcal pneumonia (BP). A secondary aim was to establish the potential coverage in Denmark of currently licensed PCVs should they prove efficacious in preventing adult pneumococcal pneumonia.

## Methods

### Study population

From January through December 2011 consecutive first episodes of pneumococcal pneumonia in patients over the age of 15 years with a pneumococcal isolate from the lower respiratory tract and/or blood diagnosed at one of 15 hospitals served by one of three Departments of Clinical Microbiology at Aalborg, Herlev or Hvidovre Hospital, respectively, were included. The entire catchment population was approximately 1.9 million adults (16 years or older), serving patients from the 15 hospitals and over 1000 general practitioners. The National Health Board (record no. 7-604-04-2/332) and the Danish Data Protection Agency (record no. 2011-41-6426) approved this study. Informed consent is not required by Danish legislation for register-based studies.

Clinical, demographic, laboratory and microbiological data were abstracted from patient charts.

Demographic data included age, sex, smoking, alcohol use, and pneumococcal vaccination status. We ascertained the presence of acute respiratory symptoms (dyspnoea, cough, sputum production or chest pain) and recorded chest radiograph findings. Plasma C-reactive protein level (CRP), white blood cell count (WBC), body temperature, mean arterial blood pressure (MAP), peripheral oxygen saturation (SAT), and two disease severity scores, the CURB-65 score [17] and the Pitt score [18] within 24 hours of pneumonia was noted. Nosocomial infection was defined as an infection occurring more than 48 hours after admission to hospital [19].

Charlson’s comorbidity index (CCI) scores were calculated for each patient. The CCI score is a weighted index that includes 19 different disease categories and takes into account the number and the seriousness of comorbid disease [20,21]. Data on each patient’s complete hospitalization history of co-existing diseases was obtained from The Danish National Registry of Patients (DNRP) using linkage by unique civil registration numbers. The DNRP includes all patient admissions to Danish hospitals since 1977 and all hospital outpatient visits since 1995 [22]. Three comorbidity levels were defined: low (score of 0), medium (1,2), and high (≥3).

Microbiological data included the results for all sputum Gram-stains, cultures from the lower airways, blood cultures, and pleural fluid cultures as obtained at the managing physician’s discretion (e.g. when infection was suspected). The methods for performing each of these tests were those in place at the participating institutions, however, at all departments sputum was processed for culture only if it contained a significant number of leukocytes (>25 per low power field) with or without columnar epithelial cells, and/or no or few squamous epithelial cells (<10 per low power field) [23]. Blood samples were cultured for at least five days before declared negative. A sample was only regarded as positive for pneumococci if a culture isolate was available. Identification of *S. pneumoniae* was done with standard methods including Gram stain, detection of capsular antigen by latex agglutination as well as optochin test or bile solubility test [24]. All isolates were serotyped by pneumotest latex and Quellung reaction using type-specific pneumococcal rabbit-antisera at Statens Serum Institut (SSI) as previously described (SSI-Diagnostica, Copenhagen, Denmark) [24,25].

### Definition of pneumonia

We defined pneumonia as a new infiltrate on a chest radiograph together with two or more clinical symptoms (dyspnoea, cough, sputum production, chest pain, and/or body temperature > 38°C or < 36.1°C) and/or WBC > 10^9^ cells/L) or plasma CRP > 30 mg/L) [26]. Severe pneumonia was defined as pneumonia with a CURB-65 score ≥ 2 [17].

### Statistics

All values are median and interquartile range or percentages with 95% confidence interval (CI) unless otherwise specified. Differences in continuous variables were tested by the Mann-Whitney test. A difference in categorical data was tested by Fisher’s exact test or χ^2^ statistics, as appropriate. We used logistic regression to analyze factors associated with infection with a PCV serotype. Data was analyzed using IBM SPSS Statistics (version 20; IBM, Armonk, New York, USA).

## Results

### Subjects

During 2011, 1101 consecutive individual subjects suspected of pneumonia and with a pneumococcal culture isolate were included in the study. Six hundred and twenty-nine were excluded because they did not fulfill the criteria for pneumonia or bacteremia; 385 did not have a chest radiographic infiltrate, 211 had not had a chest radiograph performed; and 43 could not be cultured at SSI. Of the 472 included individuals with a radiographic infiltrate, 280 had *S. pneumoniae* isolated from an airway sample and clinical and laboratory criteria compatible with bacterial pneumonia (NBP). Eight had a different etiology by blood culture and were excluded (*Staphylococcus aureus* (3), *S. epidermidis* (1), *Haemophilus influenzae* (1), non-hemolytic streptococcus (1), and *Fusobacterium* sp. (1)). Of the remaining 272, 199 (73%) had a negative blood culture and 73 did not have a blood culture. Patients without a blood culture were comparable with regard to sex and comorbidity but had lower temperature (P=0.0001), WBC (P=0.04), plasma CRP (P=0.0001), Pitt score (P=0.001), CURB-65 score (P=0.04) and higher MAP (P=0.002) compared to patients with a blood culture. One hundred and ninety-two had an infiltrate and *S. pneumoniae* isolated from blood (BP). For NBP, 248 of 272 (91%) cases were community-acquired and 181 of 192 (94%) BP cases were community-acquired. Four hundred and sixty-five (98.5%) of all included patients were hospitalized.

Individuals with NBP were of comparable age, were more often men, had more respiratory symptoms, lower WBC, lower plasma CRP, lower body temperature, lower severity scores and more comorbidity compared to individuals with BP. Comorbidity among NBP was predominantly chronic pulmonary disease. There were no significant differences between groups with regard to smoking, alcohol use, travel history or pneumococcal immunization. Characteristics of study subjects are given in table 1.

**Table 1:** Characteristics of cases with non-bacteremic and bacteremic pneumococcal pneumonia.

	Non-bacteremic pneumonia, N=272	Bacteremic pneumonia, N=192	P value
**Age, years**	68 (58-78)	68 (56-80)	0.82
**Male**	172 (63.2%)	107 (55.7%)	0.12
**Respiratory symptoms**			
0	3 (1%)	8 (4%)	
1	6 (2%)	10 (5%)	
2	22 (8%)	29 (15%)	
≥3	128 (47%)	51 (27%)	
Unknown	113 (42%)	94 (50%)	0.0001
**Blood white blood count, 10^9^/L**	14.3 (10.9-18.8)	16.9 (12.1-22.0)	0.001
**Plasma C-reactive protein, mg/L**	163 (68-249)	324 (218-406)	0.0001
**Temperature,°C**	38.0 (37-38.8)	38.6 (37.5-39.2)	0.0001
**Mean arterial pressure, mmHg**	88 (73-99)	79 (70-97)	0.009
**Peripheral oxygen saturation, %**	92 (88-96)	93 (89-95)	0.71
**CURB-65 score**	1 (1-2)	2 (1-3)	0.003
**Pitt score**	0 (0-2)	1 (0-2)	0.014
**Charlson Comorbidity Index score**			
Low (0)	74 (27%)	78 (40%)	
Medium (1,2)	103 (38%)	57 (30%)	
High (≥3)	95 (35%)	57 (30%)	0.009
Median score (IQR)	2 (0-3)	1 (0-3)	0.06
**Chronic pulmonary disease**	120 (44%)	38 (20%)	0.0001
**Cerebrovascular disease**	53 (20%)	23 (12%)	0.04
**Chronic heart disease**	40 (15%)	27 (14%)	0.85
**Diabetes**	29 (11%)	29 (15%)	0.15
**HIV**	2 (0.7%)	2 (1.0%)	0.73
**Liver disease**	12 (4%)	8 (4%)	0.89
**Malignancy**	49 (18%)	42 (22%)	0.30
**Smoking**			
Never	20 (7%)	30 (16%)	
Former	79 (30%)	50 (27%)	
Current	151 (56%)	93 (49%)	
Unknown	19 (7%)	15 (8%)	0.03
**Alcohol use**			
No	87 (32%)	76 (40%)	
Moderate	90 (33%)	50 (27%)	
High	61 (23%)	32 (17%)	
Unknown	32 (12%)	30 (16%)	0.08
**Travel history**			
No	139 (51%)	90 (47%)	
Yes	8 (3%)	6 (3%)	
Unknown	125 (46%)	96 (50%)	0.81
**Pneumococcal immunization**			
No	81 (30%)	68 (35%)	
Yes	1 (0.3%)	1 (0.5%)	
Unknown	190 (70%)	123 (64%)	0.33

All values are median and interquartile range or frequency with percentage.

### Serotypes

**Figure 1:**
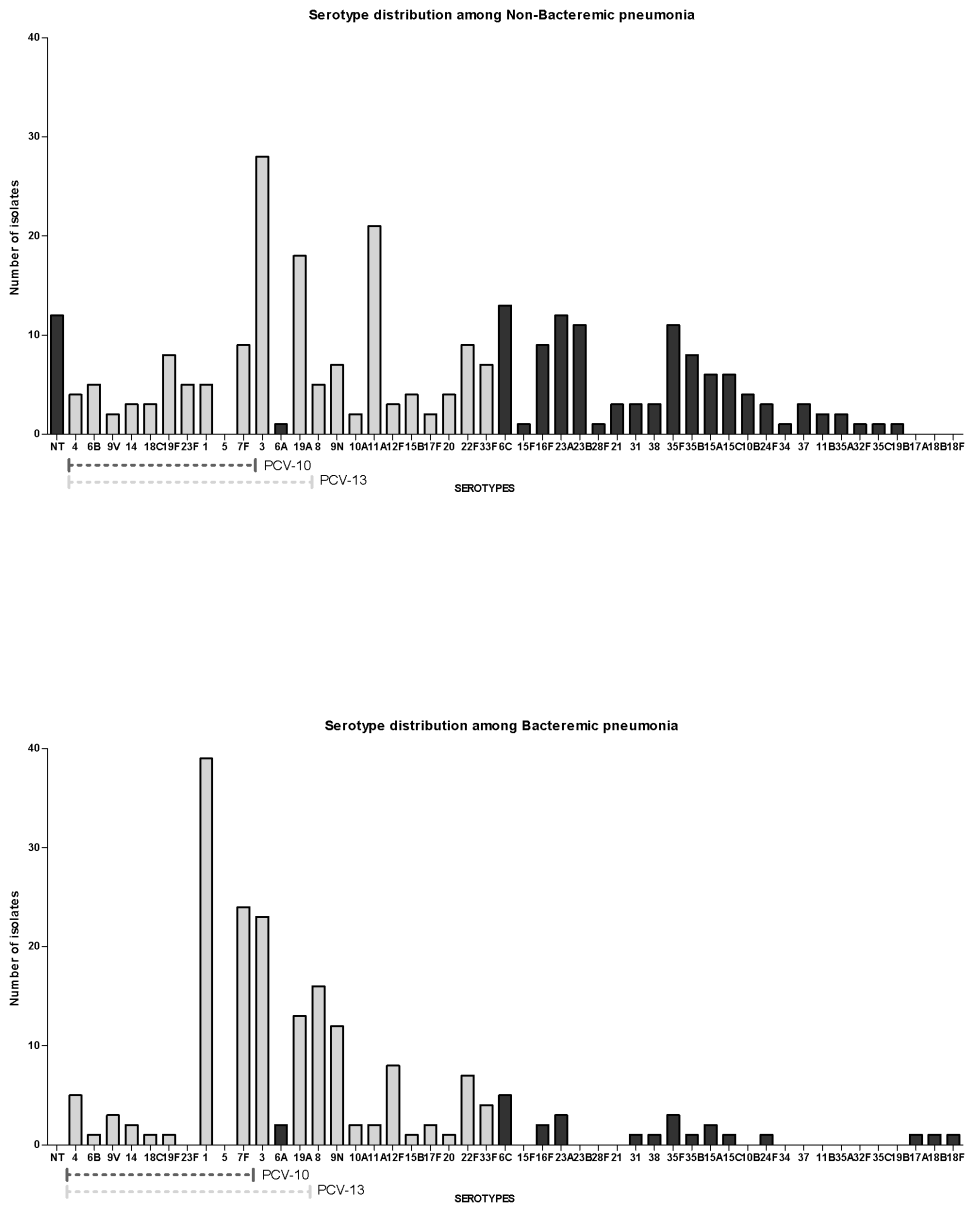
Serotype distribution for non-bacteremic (top panel) and bacteremic pneumococcal pneumonia (lower panel). Serotypes are listed starting with the non-typeable (NT), followed by serotypes in the pneumococcal conjugate vaccine (PCV)-10 and PCV-13, and then the remaining serotypes. Light gray bars represent serotypes included in the 23-valent pneumococcal polysaccharide vaccine.

In total, 46 different pneumococcal serotypes were identified. In NBP, 5 of 43 different serotypes (3, 11A, 19A, 6C, and 7F) accounted for nearly a third of isolates. Among BP cases, 5 of 34 serotypes accounted for two-thirds of isolates (1, 7F, 3, 8 and 19A). The serotype distribution is shown in table 2 and Figure 1.

**Table 2:** Pneumococcal serotype distribution.

Serotype	Non-bacteremic pneumonia, N=272 (%)	Bacteremic pneumonia, N=192 (%)
3**	28 (10.3)	23 (12.0)
11A	21 (7.7)	2 (1.0)
19A**	18 (6.6)	13 (6.8)
6C	13 (4.8)	5 (2.6)
23A	12 (4.4)	3 (1.6)
NT	12 (4.4)	0
23B	11 (4.0)	0
35F	11 (4.0)	3 (1.6)
7F*	9 (3.3)	24 (12.5)
16F	9 (3.3)	2 (1.1)
22F	9 (3.3)	7 (3.6)
19F*	8 (2.9)	1 (0.5)
35B	8 (2.9)	1 (0.5)
9N	7 (2.6)	12 (6.2)
33F	7 (2.6)	4 (2.1)
15A	6 (2.2)	2 (1.0)
15C	6 (2.2)	1 (0.5)
1*	5 (1.8)	39 (20.3)
6B*	5 (1.8)	1 (0.5)
8	5 (1.8)	16 (8.3)
23F*	5 (1.8)	0
4*	4 (1.5)	5 (2.6)
10B	4 (1.5)	0
15B	4 (1.5)	1 (0.5)
20	4 (1.5)	1 (0.5)
12F	3 (1.1)	8 (4.2)
14*	3 (1.1)	2 (1.0)
18C*	3 (1.1)	1 (0.5)
21	3 (1.1)	0
24F	3 (1.1)	1 (0.5)
31	3 (1.1)	1 (0.5)
37	3 (1.1)	0
38	3 (1.1)	1 (0.5)
9V*	2 (0.7)	3 (1.6)
10A	2 (0.7)	2 (1.0)
11B	2 (0.7)	0
17F	2 (0.7)	2 (1.0)
35A	2 (0.7)	0
6A**	1 (0.4)	2 (1.0)
15F	1 (0.4)	0
19B	1 (0.4)	0
28F	1 (0.4)	0
32F	1 (0.4)	0
34	1 (0.4)	
35C	1 (0.4)	0
17A	0	1 (0.5)
18B	0	1 (0.5)
18F	0	1 (0.5)

* PCV10 & 13

** PCV13 only

NT: non-typeable

### Pneumococcal conjugate vaccine coverage

The serotypes included in current pneumococcal protein conjugate vaccine formulations are: serotypes 1, 4, 5, 6B, 7F, 9V, 14, 18C, 19F, 23F in the ten-valent pneumococcal *Haemophilus influenzae* protein D conjugate vaccine (PCV10; GlaxoSmithKline) and serotypes 3, 6A, and 19A are additionally included in the 13-valent pneumococcal conjugate vaccine (PCV13; Pfizer). PPV23 includes 1, 2, 3, 4, 5, 6B, 7F, 8, 9N, 9V, 10A, 11A, 12F, 14, 15B, 17F, 18C, 19F, 19A, 20, 22F, 23F and 33F. PCV10 and -13 covered 16% (95% confidence interval (CI): 11-21%) and 34% (95% CI: 25-43%) of NBP isolates, respectively. PCV10 and -13 covered 40% (95% CI: 32-48%) and 59% (95% CI: 48-70%) of BP isolates, respectively. PPV23 covered 57% (95% CI: 48-66%) of NBP and 87% (95% CI: 74-100%) of BP cases. The difference in coverage between NBP and BP cases was statistically significant for PCV10 (P=0.0001), PCV13 (P=0.0001) and PPV23 (P=0.0001).

In order to identify possible clinical correlates associated with infection caused by a vaccine serotype, we performed multivariate logistic regression analysis, Infection with a PCV10 or a PCV13 vaccine serotype was more likely for bacteremic cases and cases with higher plasma CRP values. Infection with PCV10 vaccine types was less likely with increasing CCI score while this was not the case for PCV13 vaccine types (Table 3).

**Table 3:** Factors associated with infection with a pneumococcal conjugate vaccine serotype.

	Adjusted* OR (95% CI)	P value
**PCV10 serotype**		
**Bacteremia**		
No	1.0	
Yes	2.72 (1.51-4.90)	0.001
**Plasma C-reactive protein, per mg/L increment**	1.003 (1.001-1.005)	0.003
**Charlson Comorbidity Index score**		
Low (0)	1.0	
Medium (1,2)	0.66 (0.33-1.34)	0.25
High (≥3)	0.45 (0.20-0.98)	0.04
**PCV13 serotype**		
**Bacteremia**		
No	1.0	
Yes	1.88 (1.13-3.13)	0.02
**Plasma C-reactive protein, per mg/L increment**	1.004 (1.002-1.005)	0.0001

OR: odds ratio; CI: confidence interval; PCV: pneumococcal conjugate vaccine

* Variables included in the model: age, sex, CURB-65, Pitt score, temperature, mean arterial blood pressure, peripheral oxygen saturation, white blood cell count, C-reactive protein and chronic pulmonary disease.

### Serotype and disease severity

Highly invasive serotypes are defined as 1, 5, and 7F [12,27]. Serotype 5 was, however, not represented in our population. Fourteen of 272 (5%) cases of NBP were caused by invasive serotypes compared to 63 of 192 (33%) of BP cases (P=0.0001). Invasiveness did not correlate with age group for NBP cases (P=0.73) but there were a higher proportion of invasive cases in the youngest age group with bacteremic pneumonia (P=0.001, table 4). For NBP, invasive serotypes were associated with disease severity; individuals with Pitt score > 3 were more often infected with an invasive serotype (5 of 31 (16%) vs. 8 of 230 (3%), P=0.01) compared to Pitt score ≤ 3. Similarly, there was a trend towards an association between invasive serotypes and higher CURB-65 score; individuals with CURB-65 > 3 were more often infected with invasive serotypes (5 of 54 (9%) vs. 7 of 204 (3%), P=0.07) compared to CURB-65 ≤ 2. Comorbidity was not associated with invasive serotypesfor NBP (P=0.87). In contrast, for BP a high Pitt or CURB-65 score was associated with a non-invasive serotype; Pitt score > 3: 4 of 28 (14%) vs. Pitt score ≤ 3: 59 of 163 (36%), P=0.02; and CURB-65 score > 2: 61 of 188 (20%) vs. CURB-65 ≤ 2: 50 of 132 (38%), P=0.02. Similarly, there was an inverse relationship between CCI score and serotype invasiveness: 37 of 78 (48%) episodes in patients with low CCI; 18 of 57 (32%) and 8 of 57 (14%) with high CCI were caused by invasive serotypes (P=0.001).

**Table 4:** Age distribution and serotype invasiveness.

	Non-bacteremic pneumonia		Bacteremic pneumonia	
Age group, years	Invasive serotype, n=14	Non-invasive serotype, n=258	Invasive serotype, n=63	Non-invasive serotype, n=129
16-49	2 (14%)	31 (12%)	23 (37%)	14 (11%)
50-64	3 (22%)	67 (26%)	14 (22%)	32 (25%)
65-74	6 (43%)	70 (27%)	11 (17%)	31 (24%)
75-84	2 (14%)	65 (25%)	8 (13%)	30 (23%)
85+	1 (7%)	25 (10%)	7 (11%)	22 (17%)

Invasive serotypes: 1 and 7F

Based on previously observed specific serotype associations with risk of mortality in invasive pneumococcal disease [10], serotypes were predefined as high-risk: 3, 10A, 11A, 15B, 16F, 17F, 19F, 31, and 35F; moderate-risk: 6A, 6B, 9N, 12F, 14, 18C, 19A, 20, 23A, 23F, and 24F; and low-risk: 1, 4, 5, 7F, 8, 9V, 22F, 33F and 38 [10]. For NBP, there were no associations between serotype risk group and age (P=0.81), CCI score (P=0.98), CURB-65(P=0.32) or Pitt score (P=0.58).

For BP cases there was no association with age group (P=0.19) but there was a positive association between serotype risk group and comorbidity: 9 of 78 (12%) with low CCI; 11 of 57 (19%) with medium CCI; and 17 of 57 (30%) with high CCI were infected with a high-risk serotype compared to 50 of 77 (64%) with low CCI; 27 of 57 (47%); and 22 of 57 (39%) with high CCI being infected with a low risk serotype (P=0.02). High-risk serotypes were more frequent with disease severity: 27 of 163 (17%) with low Pitt score and 9 of 28 (32%) with high Pitt score were infected with a high-risk serotype compared to 91 of 163 (56%) and 8 of 28 29%) with a moderate or low risk serotype (P=0.02).

## Discussion

In this study, we show that the serotype distribution of non-bacteremic and bacteremic pneumococcal pneumonia differ substantially. Further, we show that PCV coverage would be low for non-bacteremic pneumococcal pneumonia and acceptable for bacteremic pneumococcal pneumonia.

Preliminary findings from the on-going Dutch randomized clinical trial designed to evaluate the efficacy of PCV13 against adult community-acquired pneumonia are anticipated in 2014 [28]. In their trial design they have estimated a PCV13 coverage rate that is 10-15% higher for IPD than our study suggesting that the findings from The Netherlands may have to be adjusted to country-specific coverage rates.

Only two other studies have reported on the serotype distribution in non-bacteremic pneumococcal pneumonia [15,16]. The two studies and ours each applied slightly different definitions of pneumonia and determined serotypes using different methodology. Although our study applied the strictest definition of pneumonia, we believe some comparisons can be made. Similar to us, Domenech et al. showed a wider diversity of serotypes in non-bacteremic than in bacteremic cases. The distribution of serotypes between Denmark and Spain differed; while two (3 and 11A) of the 3 most frequent serotypes were similar just 4 (19A and 23A additionally) serotypes among the 10 most frequent were shared. A large proportion of our patients had chronic obstructive lung disease. The distribution of serotypes showed a similar pattern when compared to the Spanish patients with chronic obstructive lung disease, i.e. 3 and 11A were the most frequent serotypes and 4 of the top 10 most frequent were similar (19A and 6C additionally) [15]. Bewick et al. did not clearly distinguish between non-bacteremic and bacteremic cases, thus, making comparisons between the studies difficult. Regardless, looking at the top 10 serotypes, only 4 were similar across studies. This clearly implies that there is geographical variation in the serotype distribution of non-bacteremic pneumonia similar to the known geographical variation of bacteremic pneumococcal disease. Part of the differences in distribution may be caused by antibiotic selection of specific serotypes dependent on the different rates of *S. pneumoniae* resistance between serotypes [29]. Interestingly, 11A and 23A were common non-bacteremic serotypes in Denmark and Spain but neither serotype was detected in England. So while the addition of these two serotypes to current PCVs would increase the coverage by 10% in some countries it would add nothing in other countries.

Serotypes differ in their age distribution and studies suggest that PCV serotypes are more prevalent in the elderly adults compared to non-elderly adults [12,13]. Thus, it is possible that adult age groups may benefit differentially from PCVs. This was suggested by results from Bewick et al that showed an association between PCV serotypes and age in pneumococcal pneumonia [16]. However, we were unable to confirm any association between age and vaccine serotypes for neither PCV10 nor PCV13. We did, however, show that younger individuals were more likely to be infected with an invasive serotype suggesting that younger, and presumably healthier, individuals are resistant to the less invasive or opportunistic serotypes.

Comorbidity has been suggested to increase susceptibility to less invasive serotypes also known as opportunistic serotypes [12]. Higher comorbidity burden was associated with a reduced likelihood of infection with a PCV10 serotypes but not with a PCV13 serotype. This appears to be explained by the fact that PCV13 includes three additional serotypes that are considered as less invasive and that are prevalent among cases of non-bacteremic pneumonia.

Serotype invasiveness affected disease severity differentially for non-bacteremic and bacteremic pneumococcal pneumonia. Infection with invasive serotypes in non-bacteremic cases lead to more severe disease as assessed by both pneumonia and sepsis severity scores. In contrast, bacteremic cases had lower indices of disease severity associated with invasive serotypes. Comorbidity did not account for this in non-bacteremic cases since there was no association with CCI score and serotype invasiveness whereas there was an inverse relationship between CCI score and serotype invasiveness for bacteremic pneumonia. Serotypes associated with high mortality did not affect disease severity in non-bacteremic pneumonia. Individuals with bacteremic pneumonia with low comorbidity burden more often were infected by high-risk serotypes and conversely. This may suggest that individuals with comorbidity are more susceptible to infection with less virulent serotypes. Disease severity of bacteremic pneumonia was higher for individuals infected with high-risk serotypes compared to low-risk serotypes.

The strength of this study is the population-based design and careful ascertainment of pneumonia. There are several potential limitations of our study. Blood culture was only performed in three-quarter of patients with NBP. However, when we excluded patients without blood culture from the analysis the serotype coverage rate remained low (29%). We did not have information on pre-hospital antibiotic use. Potentially, resistant serotypes could be differentially selected among patients prescribed antibiotics prior to being admitted to hospital. Antibiotic resistance among *S. pneumoniae* is low in Denmark. Of 901 isolates tested in the DANMAP program less than 5% were resistant to penicillins or macrolides [30]. The 43 penicillin non-susceptible *S. pneumoniae* in 2011 belonged to 10 different serotypes, and the most commonly found serotypes were type 19A (46.5%), 15A (16.3%) and 6C (9.3%). Similarly, the 44 macrolide resistant *S. pneumoniae* from 2011 belonged to 11 different serotypes and the most commonly found serotypes were type 19A (36.4%), 15A (20.5%) and 6C (15.9%). This would suggest that, although pre-hospital antibiotics could select for specific serotypes, the bias would be very low. More patients with NBP had chronic obstructive lung disease compared to patients with BP. We speculate that this may reflect a lower threshold for investigation and sputum sampling of patients with known chronic lung disease.

In conclusion, serotype diversity was greater in NBP than BP cases and more likely to be caused by a less invasive serotype. Thus, the potential coverage of currently licensed PCVs was low, and, therefore, a vaccine constructed to prevent pneumococcal pneumonia in adults should include more serotypes to offer sufficient protection. However, a proper controlled clinical trial is required to assess the efficacy of PCVs in adult populations.
